# Tricuspid Transcatheter Edge-to-Edge Repair at a Crossroads: Prognosis-Shaping Intervention or High-Tech Palliation?

**DOI:** 10.3390/jcm15020443

**Published:** 2026-01-06

**Authors:** Andreas Mitsis, Marios Ioannides, Christis Rotos, Nikolaos P. E. Kadoglou, Christos Eftychiou

**Affiliations:** 1 Cardiology Department, General Hospital of Nicosia, Limassol Old Road No. 215, 2029 Nicosia, Cyprus; mariosioannides1969@gmail.com (M.I.); christisrotos@yahoo.com (C.R.); chr6eft@gmail.com (C.E.); 2 Medical School, University of Cyprus, 2029 Nicosia, Cyprus; nikoskad@yahoo.com

**Keywords:** tricuspid regurgitation, transcatheter edge-to-edge repair, right ventricular failure, structural heart disease, tricuspid valve interventions, heart failure, patient selection

## Abstract

Tricuspid regurgitation (TR) has historically been undertreated despite its strong association with morbidity and mortality. Surgical correction of isolated TR is not routinely performed and has shown limited survival benefit, leaving a substantial unmet need for minimally invasive therapies. Transcatheter edge-to-edge repair (T-TEER) has emerged as a promising therapeutic option for patients with symptomatic severe or greater (≥severe) TR who are unsuitable for surgery. Recent randomized trials, including the TRILUMINATE Pivotal and the Tri-FR study, and real-world registries consistently demonstrate significant improvements in TR severity, functional status, and quality of life following T-TEER. Although benefits in hard clinical endpoints such as mortality or heart failure hospitalizations remain less conclusive, growing evidence suggests potential prognostic advantage in selected patients, particularly those with preserved or mid-range right ventricular function. Anatomical suitability, RV performance, and optimized patient selection are crucial determinants of success. As ongoing large-scale trials continue to evaluate long-term outcomes, T-TEER currently occupies a therapeutic space between palliative intervention and disease-modifying therapy, providing substantial symptom relief with the potential for broader clinical benefit. This review summarizes current evidence, patient selection strategies, and perspectives on the evolving role of T-TEER in the management of severe TR.

## 1. Introduction

Tricuspid regurgitation (TR) has traditionally been referred to as the “forgotten valve disease”, receiving far less attention and treatment consideration than left-sided valvular conditions despite its significant clinical impact [1]. Contemporary imaging and epidemiologic research demonstrate that severe TR leads to progressive right ventricular (RV) dysfunction, systemic venous congestion, impaired functional capacity, and increased mortality [2]. Medical therapy alone provides symptomatic relief but does not modify disease progression, and isolated surgical repair remains infrequent due to high procedural risk and lack of proven long-term benefit [3,4]. This therapeutic gap has seemed to be filled by the development of transcatheter tricuspid valve interventions (TTVI), particularly transcatheter edge-to-edge repair (T-TEER), which has rapidly evolved into the leading percutaneous option for severe symptomatic TR. Throughout this review, TR severity is discussed in accordance with contemporary expanded grading schemes, and the presented evidence primarily applies to patients with severe or greater (≥severe) TR, including massive and torrential regurgitation where specified [5,6].

Despite the rapid expansion of TTVI and the growing adoption of T-TEER, important uncertainties remain regarding its role beyond symptomatic relief. While randomized trials and real-world registries consistently demonstrate improvements in tricuspid regurgitation severity, functional status, and quality of life, evidence supporting a definitive prognostic benefit remains inconclusive. In particular, the optimal timing of intervention, the identification of patient subgroups most likely to derive prognostic benefit, and the influence of right ventricular function and disease stage on outcomes are not fully established. This review aims to address these knowledge gaps by integrating data from randomized controlled trials, large observational registries, and disease-stage and right ventricular function–stratified analyses. By synthesizing contemporary evidence across these complementary sources, we seek to provide a unified interpretive framework to clarify when T-TEER should be viewed as a predominantly palliative intervention and when it may exert disease-modifying effects in carefully selected patients.

## 2. Pathophysiology and Clinical Burden of Severe TR

Functional tricuspid regurgitation (FTR) most commonly develops because of right-sided cardiac remodeling rather than primary valvular pathology. The initiating mechanism typically involves annular dilation and leaflet tethering, which comes secondary to a variety of conditions that impose chronic volume or pressure overload on the right heart [7]. Among these, atrial fibrillation, right atrial enlargement, pulmonary hypertension, and left-sided heart disease such as heart failure with preserved or reduced ejection fraction and severe mitral valve disease, play central roles in changing tricuspid valve geometry [8].

Two major phenotypes of FTR have been increasingly recognized. Atrial Functional TR (AFTR), driven primarily by isolated right atrial dilation and remodeling in the setting of long-standing atrial fibrillation. In this phenotype, the tricuspid leaflets are structurally normal, but significant annular dilation prevents adequate leaflet coaptation [9]. Ventricular Functional TR (VFTR), in which elevated pulmonary pressures and chronic right ventricular (RV) afterload led to RV dilation, papillary muscle displacement, and leaflet tethering. This mechanism is prevalent in pulmonary hypertension and left-sided heart disease [10]. Of note, patients with AFTR had significantly better survival as compared with patients with VFTR independently of other clinical and echocardiographic characteristics [11].

In addition, TR related to intracardiac RV leads represents a distinct and increasingly prevalent mechanism of functional TR. Trans-tricuspid pacing or defibrillator leads may interfere with leaflet motion, cause leaflet impingement or perforation, and exacerbate annular dilatation, ultimately leading to significant regurgitation [12]. Lead-related TR frequently coexists with atrial or ventricular functional mechanisms and is associated with worse clinical outcomes, underscoring the importance of careful anatomical assessment and mechanism-specific decision-making when considering transcatheter therapies [13].

**Figure 1:**
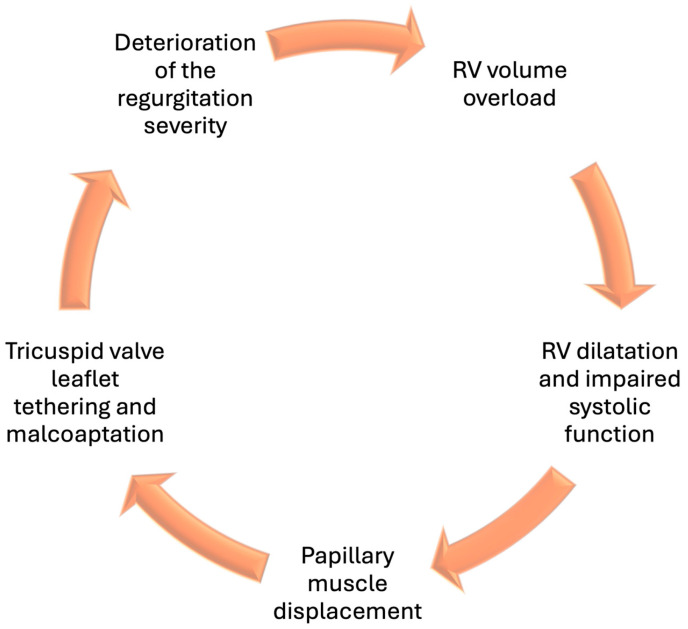
The Vicious Cycle of Functional Tricuspid Regurgitation. Functional tricuspid regurgitation progresses through a vicious cycle in which annular dilatation, leaflet tethering, and increased regurgitation promote RV dilatation, leading to RV dysfunction and worsening right-sided heart failure and congestion, which in turn further aggravate the upstream mechanisms.

Regardless of the initiating cause, progressive RV volume overload serves as the trigger for disease progression. As regurgitant volume increases, the RV undergoes abnormal remodeling characterized by dilation and gradual impairment of systolic function [Figure 1]. This worsens leaflet malcoaptation, further deterioration of the regurgitation severity in a vicious cycle that ultimately terminates in right-sided heart failure [14]. Clinically, patients present with excessive signs of systemic venous congestion including elevated jugular venous pressure, peripheral edema, ascites, and easy fatigue [15]. In advanced stages, patients may develop cardiorenal syndrome and refractory right-sided heart failure, both of which significantly complicate management.

Observational studies reveal that medically managed severe TR is associated with poor survival, underscoring the inadequacy of conservative therapy in modifying disease’s course [16]. Notably, even moderate TR has been shown to predict worse outcomes in heart failure and pulmonary hypertension cohorts [17]. These findings highlight the inadequacy of conservative therapy and the fact that diuretics may provide symptomatic relief but do not reverse underlying structural remodeling. As a result, growing emphasis has been placed on timely interventional strategies to modify the disease course before irreversible RV dysfunction develops.

## 3. Limitations of Surgical Management and the Rise of Transcatheter Therapies

**Figure 2:**
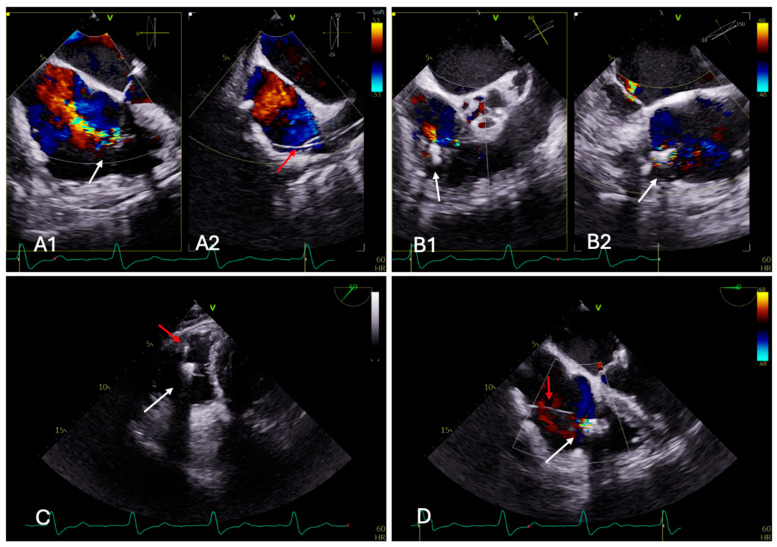
Transesophageal echocardiographic assessment of the tricuspid valve during T-TEER with a PASCAL device. These images are provided for illustrative purposes only and do not represent comparative outcome data. Panels (**A1**,**A2**). The mid-esophageal (ME) TV 4-chamber view is obtained at 0° (**A1**) and 90° (**A2**) demonstrates severe tricuspid regurgitation (TR) with a broad central jet (white arrows) (**A1**). A pacemaker lead traversing the tricuspid valve is clearly visualized (red arrow) (**A2**). Panels (**B1**,**B2**). ME RV inflow-outflow views in 60° (**B1**) and 150° (**B2**) views highlight the interaction between the trans-tricuspid pacemaker lead and the tricuspid leaflets, with focal regurgitant jets adjacent to the lead (**B1**) during the PASCAL device implantation (white arrows). Panel (**C**). Transgastric short-axis two-dimensional echocardiographic images illustrates the spatial relationship between the deployed PASCAL device (white arrow), which bridges the tricuspid leaflets, and the pacemaker lead (red arrow), coursing through the valve apparatus in the anterior–posterior commissural position without interfering with device stability or leaflet capture. Panel (**D**). Final color Doppler assessment in ME 4-chamber view confirms sustained TR reduction following TEER, with the PASCAL device (white arrow) maintaining effective leaflet coaptation despite the presence of the pacemaker lead (red arrow).

Isolated tricuspid valve surgery is not routinely practiced and has not demonstrated survival benefit in contemporary series, largely due to late referral and advanced RV dysfunction at presentation [18]. Of note, data from observational studies have shown an in-hospital mortality that reaches almost 10% after isolated TR surgery [19,20]. Interestingly, the isolated TV surgery was associated with high mortality and morbidity predicted by the severity of the presentation and not necessary by TR mechanism [20]. Consequently, most patients with significant TR remain undertreated. Transcatheter technologies, led by T-TEER, have emerged as a less invasive and more accessible alternative for high-risk individuals [Figure 2]. Until now, two primary commercial systems are available; Abbott’s TriClip (now TriClip G4/G5) and Edwards Lifesciences’ PASCAL system, with TriClip being the first FDA-approved for this purpose in the US [21], while PASCAL is also widely used and approved in other regions like Europe. Early feasibility studies have demonstrated procedural safety, high technical success, and promising short and long term clinical outcomes, prompting randomized evaluation [22,23,24,25,26].

## 4. Evidence Base for T-TEER: Clinical Trials and Real-World Data

### 4.1. Randomized Controlled Trials

a. TRILUMINATE Pivotal Trial

The TRILUMINATE Pivotal study was the first randomized controlled trial comparing T-TEER with guideline-directed medical therapy using the TriClip device [27]. The trial demonstrated significant improvements in quality of life as Kansas City Cardiomyopathy Questionnaire (KCCQ) quality-of-life score changed by a mean (±SD) of 12.3 ± 1.8 points in the TEER group, as compared with 0.6 ± 1.8 points in the control group (*p* < 0.001). Furthermore, at 30 days, 87.0% of the patients in the TEER group and 4.8% of those in the control group had TR of no greater than moderate severity (*p* < 0.001). Although mortality (9.4% in the TEER group vs. 10.6% in the control group) and annualized heart-failure hospitalization rates (0.21 events per patient-year in the TEER group vs. 0.17 events per patient-year in the control group) did not differ significantly between treatment arms, the magnitude of symptomatic improvement supports T-TEER as an effective therapy for symptom relief.

The two-year results of the study confirmed durable TR reduction, sustained gains in functional capacity, and stable safety profile [28]. Freedom from all-cause mortality, tricuspid valve surgery, and tricuspid valve intervention through 2 years was significantly higher with T-TEER compared with control (77.6% versus 29.3%; *p* < 0.0001), driven by more tricuspid valve intervention in control patients who crossed over to device treatment (3.8% versus 61.5%). Rates of all-cause mortality (17.9% versus 17.1%) and tricuspid valve surgery (2.3% versus 4.3%) were similar between groups. Moderate or less TR was present in 84% at 2 years in the device group. These findings support T-TEER as an effective therapy for symptom relief, while potential prognostic implications remain uncertain and require further investigation.

b. Tri.Fr Trial

The Tri.Fr randomized trial confirmed the symptomatic superiority of T-TEER over medical therapy, with significantly more patients achieving improvement in composite clinical outcomes at one year [29]. The trial demonstrated that adding T-TEER to optimal medical therapy (OMT) effectively reduced TR severity and led to meaningful clinical benefits, primarily reflected by improvements in patient-reported outcomes such as New York Heart Association (NYHA) functional class and the Patient Global Assessment (PGA). A favorable clinical composite response was observed in 74.1% of patients treated with T-TEER  +  OMT, compared with 40.6% of those receiving OMT alone. Rates of death (0.1% in the OMT-alone group vs. 0.03%, in the T-TEER  +  OMT group) or heart failure hospitalization (13.5% in the OMT-alone group vs. 15%, in the T-TEER  +  OMT group) were not significantly different between groups, although the absolute event numbers trended toward benefit with the T-TEER strategy. Quality-of-life gains were durable at 1 year and consistently observed across multiple measures, including NYHA class, PGA, and the KCCQ, underscoring that T-TEER  +  OMT yields a substantial and sustained improvement in health status for patients with severe, symptomatic TR. The procedure demonstrated a favorable safety profile, with a low intrahospital adverse event rate of 8.0%, including a 5.2% incidence of single-leaflet device attachment (Table 1).

### 4.2. Real-World Evidence

a. Transatlantic TRILUMINATE Registry

The Transatlantic TRILUMINATE Registry is a large, real-world, multicenter registry collecting outcomes of patients undergoing T-TEER with the TriClip system across Europe and the United States. The registry complements the RCT by offering broader, non-randomized outcomes that support and contextualize the trial’s findings. Expanded evaluation of the cohort revealed that approximately 60% of patients achieved sustained TR of no more than moderate severity at two years. RV and annular reverse remodeling improved six-minute walk distance, and reduced annualized heart-failure hospitalizations suggest possible structural and functional benefits that go beyond symptom relief. The study showed low rates of cardiovascular mortality (15.3%) and all-cause mortality (18.7%) at 2 years, while all-cause hospitalization rate decreased from 1.30 events per patient-year 1 year before device implantation to 0.66 events per patient-year 2 years after the TriClip procedure, representing a reduction of 49% (*p* < 0.0001) [30].

b. The bRIGHT Registry

In one of the largest registries to date, the bRIGHT (Observational Real-World Study Evaluating Severe Tricuspid Regurgitation Patients With the Abbott TriClip Device) study reported a 99% procedural success rate, robust TR reduction, and dramatic improvements in NYHA class and quality of life even in elderly patients with torrential TR. These findings confirm the strong translational applicability of T-TEER [31]. One-year findings from the bRIGHT study further reinforce the safety and durable effectiveness of the T-TEER in reducing TR severity among real-world patients with symptomatic TR. The study demonstrates a significant and persistent reduction in TR at 1 year, accompanied by continued low rates of adverse events and sustained improvements in quality of life, as reflected by gains in KCCQ scores (19 ± 26-point improvement, *p* < 0.00) across a broad, heterogeneous population and among centers with varying procedural experience. Notably, this analysis provides the first evidence that effective TR reduction with T-TEER attenuates the mortality risk traditionally associated with baseline TR severity. All-cause mortality per Kaplan–Meier analysis was 15.1% at 1 year for the full bRIGHT cohort. Instead, mortality correlated with the presence of greater-than-moderate residual TR. Survival was significantly lower at 1 year in subjects with TR severe or higher at 30 days (70.6%; *p* < 0.0001 overall) compared with subjects with moderate (91.9%; *p* < 0.0001), mild (88.2%, *p* = 0.0003), or trace TR (84.8%, *p* = 0.03). These findings suggest an association between successful TR reduction after T-TEER and improved survival, without establishing causality [32].

c. TriValve Registry

The TriValve registry was established to evaluate the distribution of various TTVIs including T-TEER, annuloplasty devices, leaflet and coaptation devices and valve replacement, both in the heterotopic and the orthotropic positions. The purpose of the registry was to investigate patient characteristics and initial clinical results [33]. Propensity-matched comparisons of the T-TEER population from the TriValve registry indicate lower mortality and fewer heart-failure hospitalizations in TTVI-treated patients compared with medical therapy. 1-year mortality was 23% in the TTVI group vs. 36% in the control group (*p* = 0.001) and heart failure rehospitalization was 26% in the TTVI group vs. 47% in the control group (*p* < 0.0001). Procedural success defined by achieving TR < 3+, was strongly associated with survival, highlighting the importance of anatomical suitability and technical performance and this benefit persisted after multivariable adjustment (HR ≈ 0.35–0.39) [34].

## 5. Role of RV Function and Comorbidities in Predicting Outcomes

Several observational studies underline the importance of RV performance in determining the clinical impact of T-TEER. Schlotter et al. demonstrated that patients with mid-range RV function (defined as TAPSE 13–17 mm) derive the most consistent survival benefit, whereas those with severely impaired RV function primarily achieve symptomatic relief without clear prognostic advantage [35]. Overall, 1-year mortality was 13.1% in T-TEER group vs. 25.8% in the control group (*p* = 0.031). Only patients with mid-range RV function appear to be more likely to experience favorable survival outcomes following T-TEER (p log-rank 0.004). More recently, the EuroTR Investigators analyzed 1885 patients with significant TR, including 585 conservatively treated individuals and 1300 patients who received T-TEER. Patients were stratified into early, intermediate, or advanced disease stages. Staging was determined using a composite assessment of left and right ventricular function, renal function, and natriuretic peptide concentrations, and the classification was subsequently validated in an external cohort. Of the 1885 patients included, 395 (21%) were classified as early stage, 1173 (62%) as intermediate stage, and 317 (17%) as advanced stage. Among those in the early and advanced stages, mortality did not differ between patients treated interventional and those managed conservatively (early stage: HR 0.78; 95% CI 0.34–1.80; *p* = 0.54; advanced stage: HR 1.06; 95% CI 0.71–1.60; *p* = 0.78). In contrast, percutaneous treatment was associated with lower mortality in patients with intermediate-stage disease (HR 0.73; 95% CI 0.52–0.99; *p* = 0.03) [36]. Apart from the RV status, the degree of systolic function of the left ventricle seems equally important. Kresoja et al. showed that patients with heart failure with preserved ejection fraction (HFpEF) appear to benefit more than those with HF with reduced EF (HFrEF) in terms of hospitalization and mortality reduction [37]. Procedural success was 86% in patient with HFpEF vs. 78% in patients with HFrEF. The primary endpoint occurred in 30% of HFpEF group vs. 50% of HFrEF group (*p* = 0.016) with no significant survival benefit (HR 0.53, *p* = 0.27) in HFrEF group. These findings support early tricuspid intervention before irreversible LV dysfunction occurs.

## 6. Anatomical Predictors and Patient Selection

Taking all the above into consideration, effective patient selection is essential for maximizing T-TEER outcomes (Table 2). Apart from clinical characteristics and the status of left and right ventricle, favorable anatomical characteristics are paramount in the prediction of the optimal result. Among them, smaller right atrial volumes, the presence of pacemaker lead in non-commissural position, limited leaflet tethering, and adequate coaptation tissue are the most important [38]. A recent study showed that a large coaptation gap (>7–10 mm) and non-anteroseptal location of the TR jet have been associated with procedural failure. According to the same study, T-TEER procedural success was associated with longer freedom from death and heart failure hospitalization (hazard ratio: 0.20 [95% confidence interval: 0.08 to 0.48]; *p* < 0.01), irrespective of concomitant mitral regurgitation [39].

In view of the ideal patient selection to achieve optimal survival benefit, it has been proposed that there might be limited generalizability of TRILUMINATE inclusion and exclusion criteria to real-world conditions. Stolz et al. studied the profile of large cohort of patients who underwent T-TEER at five European centers between 2016 and 2022. Patients were evaluated with respect to baseline clinical characteristics, survival, heart failure hospitalizations (HFH), and symptomatic improvement, assessed through NYHA functional class, a quality-of-life questionnaire, and 6 min walk distance. Among the 962 patients treated, 54.8% met TRILUMINATE eligibility criteria, exhibiting better left ventricular function and fewer comorbid conditions compared with ineligible patients. Both eligible and ineligible groups demonstrated similar reductions in TR, as well as comparable improvements in NYHA class, quality of life, and exercise capacity. However, significant differences emerged in 1-year outcomes patients meeting eligibility criteria showed higher survival (85% vs. 75%) and lower HFH rates (14% vs. 22%), despite similar rates of achieving TR ≤ 2+ at discharge (82% vs. 85%) [40].

As part of efforts to define the optimal candidates for tricuspid intervention, the TRI-SCORE offers a validated framework for assessing operative risk and pinpointing patients who are most likely to benefit from transcatheter therapies. In 466 patients, eight readily assessable clinical parameters formed a 0–12-point score, showing strong discriminatory ability (AUC 0.75–0.81) and outperforming EuroSCORE systems [41]. A total of 2413 patients with severe isolated functional TR were enrolled in TRIGISTRY (1217 conservatively managed, 551 isolated tricuspid valve surgery, and 645 transcatheter valve repair). Across the cohort, TRI-SCORE values were low in 32%, intermediate in 33%, and high in 35%; successful correction occurred in 97% of surgical and 65% of transcatheter cases. Survival declined progressively with increasing TRI-SCORE (*p* < 0.0001). In the low-risk group, both surgery and transcatheter treatment outperformed conservative care (93% and 87% vs. 79%). In intermediate-risk patients, overall survival did not differ, but successful correction conferred a significant advantage over conservative management. In high-risk patients, surgery or transcatheter therapy offered no survival benefit, indicating that early and successful intervention aids outcomes mainly in low- and selectively in intermediate-risk groups [41] (Table 3).

## 7. Current Guidelines and Future Directions

The more recent European Society of Cardiology (ESC) guidelines increasingly recognize T-TEER as an important therapeutic option for high-risk symptomatic patients. The 2025 ESC guidelines recommend T-TEER primarily for symptomatic, severe or greater (≥severe) secondary TR in patients deemed inoperable or high-risk for surgery by a Heart Team, often after OMT fails. While surgery is the preferred option for suitable candidates, T-TEER offers significant symptom and quality-of-life improvement, reducing heart failure hospitalizations, and is considered a key part of the management strategy alongside surgery for certain patients, especially those with concomitant left-sided valve disease [42].

Ongoing randomized trials will help determine whether T-TEER can achieve mortality or hospitalization reductions comparable to established left-sided transcatheter therapies. Large-scale studies such as CLASP II TR (NCT04097145) and TRICI-HF (NCT04634266) [43] will provide pivotal evidence on how T-TEER compares with OMT in diverse patient populations, focusing not only on survival and heart failure events but also on patient-centered quality-of-life outcomes. Parallel efforts, including TRACE-NL (NL81645.100.22), will refine patient selection criteria and validate procedural benefits across different European healthcare settings. These trials collectively aim to establish the first robust, randomized data foundation capable of informing guidelines and standardizing care pathways for symptomatic TR.

In addition to device-specific evaluations, broader registries and mechanistic studies are expanding the scientific understanding of T-TEER beyond valve-level outcomes. The European T-TEER Registry (NCT06307262) is expected to generate real-world insights into safety, durability, anatomical predictors of success, and long-term clinical trajectories in heterogeneous patient populations. Meanwhile, the Impact of TR Correction on the Gut–Liver Axis trial (NCT06902922) explores the systemic consequences of TR reduction, acknowledging the complex interplay between right-sided congestion and multi-organ function. Together, these initiatives signal a shift toward precision therapy, where optimal timing, tailored device selection, and improved comprehension of cardio-hepatic and cardiorenal relationships will shape the next generation of TR management (Table 4).

Ultimately, advances in imaging, procedural planning, and device technology such as transcatheter tricuspid valve replacement may further expand treatment options and refine patient selection [44]. In parallel with the rapid evolution of TTVI therapies in Western countries, several innovative devices developed in China have entered early clinical evaluation in recent years, reflecting growing global diversification in T-TEER technology. The Neoblazar™ system (Dawneo Medical Technology, Hangzhou, China) is a domestically designed TEER device. First-in-human experience with the Neoblazar™ system has demonstrated high procedural success and has supported the feasibility and safety of this novel platform in patients with severe TR [45] and further clinical evidence is anticipated from the ongoing NoTR study (NCT05497141). In addition, the DragonFly-T™ tricuspid repair system (Valgen Medical, Hong Kong, China) is currently undergoing clinical investigation in prospective studies (NCT05556460 and NCT05671640), aiming to evaluate its safety and efficacy in patients with significant TR. Collectively, these developments highlight the rapid technological progress in the field and support the shift toward a more, device-tailored approach to transcatheter tricuspid valve intervention.

Although this review focuses primarily on TEER, other TTVIs rapidly evolving and deserve consideration. These include transcatheter annuloplasty systems [46,47], orthotopic [48,49] and heterotopic transcatheter tricuspid valve replacement platforms [50,51], and coaptation devices [52]. Early clinical experience suggests that these modalities may offer complementary solutions for patients with unfavorable anatomy for T-TEER or advanced disease stages, further expanding the therapeutic options for significant TR.

## 8. Conclusions

T-TEER represents a major advance in the treatment of severe or greater (≥severe) symptomatic TR, consistently delivering substantial and durable improvements in quality of life, TR severity, and functional capacity. While definitive prognostic benefit has not been established, growing data suggest that selected patients, particularly those treated before severe RV dysfunction, may experience meaningful prognostic advantages. At present, T-TEER occupies a therapeutic role between palliative and disease-modifying intervention, with ongoing studies likely to clarify its long-term impact. As clinical experience expands, early diagnosis, optimal patient selection, and timely referral will be essential in maximizing the transformative potential of this emerging therapy.

## Figures and Tables

**Table 1:** Key Clinical Studies of Tricuspid TEER.

Study	Design/N	Device	Key Outcomes
TRILUMINATE Pivotal RCT [27]	RCT (TEER vs. OMT)/n = 350	TriClip	87% achieved TR ≤ moderate at 30 days; significant QoL improvement (KCCQ + 12.3); no difference in mortality or HFH
TRILUMINATE Pivotal—2-year [28]	RCT follow-up/n = 285	TriClip	Durable TR reduction (84% ≤ moderate); sustained functional benefit; similar mortality between groups
Tri.Fr Trial [29]	RCT (T-TEER + OMT vs. OMT)/n = 404	TEER (TriClip/PASCAL)	Higher clinical composite response (74.1% vs. 40.6%); significant symptom and QoL improvement; no mortality difference
Transatlantic TRILUMINATE Registry [30]	Prospective registry/n = 85	TriClip	~60% TR ≤ moderate at 2 years; improved functional capacity; 49% reduction in HF hospitalizations
bRIGHT Registry [31,32]	Real-world registry/n = 511	TriClip	99% procedural success; marked NYHA and QoL improvement; 1-year mortality 15.1%, driven by residual TR
TriValve Registry [33,34]	International registry/n ≈ 470	Mixed: mainly TEER	Procedural success associated with improved survival and fewer HF hospitalizations
Schlotter et al. [35]	Multicenter propensity-matched cohort/n = 684	TEER devices	Survival benefit limited to mid-range RV function (TAPSE 13–17 mm)
EuroTR Investigators [36]	Large multinational registry/n = 1885	TEER vs. conservative	Mortality benefit confined to intermediate disease stage (HR 0.73)
Kresoja et al. [37]	Cohort study/n = 111	TEER	Greater benefit in HFpEF; no survival benefit in HFrEF

HF: Heart Failure; HFrEF: Heart Failure with Reduced Ejection Fraction; HFpEF: Heart Failure with Preserved Ejection Fraction; HFH: Heart Failure Hospitalization; KCCQ: Kansas City Cardiomyopathy Questionnaire; N: number; OMT: Optimal Medical Therapy; RCT: Randomized Controlled Trial; RV: Right Ventricle; TAPSE: Tricuspid Annular Plane Systolic Excursion; TEER: Transcatheter Edge-to-Edge Repair; TR: Tricuspid Regurgitation.

**Table 2:** Determinants of T-TEER Success and Failure.

Domain	Predictor	Effect on Outcomes
Right Ventricular Function [35]	Mid-range RV function (TAPSE 13–17 mm)	Greatest survival benefit after TEER
	Severe RV dysfunction	Symptomatic benefit only; little prognostic gain
Disease Stage (EuroTR) [36]	Intermediate stage	Significant survival benefit (HR 0.73)
	Early or advanced stages	No survival advantage vs. conservative management
Left Ventricular Function [37]	HFpEF	Better clinical outcomes & HFH reduction
	HFrEF	Less benefit; no clear survival impact
Anatomy [38]	Small RA volume	Favorable
	Limited leaflet tethering	Higher procedural success
	Coaptation gap >7–10 mm	Strong predictor of failure
	TR jet non-anteroseptal	Lower success likelihood
Leads [38]	Pacing lead in non-commissural position	Favors TEER feasibility
Procedural Success [40]	Achieving TR ≤ moderate	Strongly predicts survival (HR~0.20)
Risk Scores [41]	Low/intermediate TRI-SCORE	Benefit from TEER or surgery
	High TRI-SCORE	No benefit vs. conservative therapy

HFH: Heart Failure Hospitalization; HFpEF: Heart Failure with Preserved Ejection Fraction; HFrEF: Heart Failure with Reduced Ejection Fraction; HR: Hazard Ratio; RA: Right Atrium; RV: Right Ventricle; TAPSE: Tricuspid Annular Plane Systolic Excursion; TEER: Transcatheter Edge-to-Edge Repair; TR: Tricuspid Regurgitation.

**Table 3:** Clinical Benefits vs. Palliative Effects of T-TEER.

Domain	What T-TEER Improves (Evidence)	What Remains Limited
Symptoms	Large, consistent improvements in NYHA class, PGA, QoL [27,28,29]	Not curative in advanced RV failure
Quality of Life	TRILUMINATE: KCCQ + 12.3 points; bRIGHT: +19 points [27,28]	Benefit declines with severe RV dysfunction
Functional Capacity	↑ 6MWD; improved exercise tolerance [27,28,29]	Less improvement in late-stage TR
TR Severity	Robust reduction (≥80% achieve ≤moderate TR in trials) [27,28,29]	Recurrence more common with large coaptation gaps
Heart Failure Hospitalizations	Transatlantic TRILUMINATE: almost 49% [30]	RCTs show no significant reduction vs. OMT yet
Mortality	Signal of benefit in intermediate RV disease & mid-range TAPSE [35,36]	No proven mortality reduction in RCTs
Organ Congestion (renal/hepatic)	Some reverse remodeling reported [30,31,32]	Persistent congestion in advanced disease
Overall Categorization	Disease-modifying signals in selected patients’ subgroups [35,36,37]	Primarily palliative in late-stage TR

KCCQ: Kansas City Cardiomyopathy Questionnaire; NYHA: New York Heart Association (functional class); OMT: Optimal Medical Therapy; RV: Right Ventricle; TEER: Transcatheter Edge-to-Edge Repair; TR: Tricuspid Regurgitation; ↑: increase.

**Table 4:** Ongoing Clinical Studies of Tricuspid TEER.

Study	Identifier	Design/Population	Purpose
CLASP II TR	NCT04097145	Randomized (PASCAL vs. OMT)	Evaluate impact on mortality & HF hospitalizations
TRICI-HF (DZHK-24)	NCT04634266	RCT in HF population	Assess TEER in HF with significant TR
TRACE-NL	NL81645.100.22	National trial	Refine patient selection and real-world outcomes
European T-TEER Registry	NCT06307262	Multinational prospective registry	Long-term durability, safety, anatomical predictors
Impact of TR Correction on Gut–Liver Axis	NCT06902922	Mechanistic clinical study	Evaluate systemic organ effects of TR reduction

HF: Heart Failure; NCT: National Clinical Trial (identifier); OMT: Optimal Medical Therapy; RCT: Randomized Controlled Trial; TEER: Transcatheter Edge-to-Edge Repair; TR: Tricuspid Regurgitation.

## Data Availability

All data supporting this article are available upon reasonable request.
